# Accuracy of patient dose estimation in cone beam computed tomography in breast irradiation by size‐specific dose estimates with position correction

**DOI:** 10.1002/acm2.13851

**Published:** 2022-11-30

**Authors:** Hiroyuki Ueno, Kosuke Matsubara, Sayuri Bou, Masato Hizume

**Affiliations:** ^1^ Division of Health Sciences Graduate School of Medical Sciences Kanazawa University Kanazawa Japan; ^2^ Department of Radiology Takaoka City Hospital Takaoka Japan; ^3^ Department of Radiotherapy Takaoka City Hospital Takaoka Japan; ^4^ Department of Radiology Graduate School of Medical Sciences Kanazawa University Kanazawa Japan

**Keywords:** cone‐beam computed tomography, image‐guided radiation therapy, size‐specific dose estimates

## Abstract

This study aims to investigate the effects of the position correction of size‐specific dose estimates (SSDE) on patient dose estimation in cone beam computed tomography (CBCT). The relationship between the phantom position and absorbed dose in the right breast was studied using optically stimulated luminescence dosimeters and a simulated human body phantom. The effect of position correction for CT dose index (CTDI) on SSDE was investigated in 51 patients who underwent right breast irradiation by comparing the SSDE with position correction and SSDE without position correction. The absorbed dose in the right breast tended to decrease by 10.2% as the phantom was placed away from the center of CBCT. The mean and standard deviation of SSDE were 2.54 ± 0.29 and 2.92 ± 0.30 mGy with and without position correction, respectively. The SSDE with position correction was 13.1% lower than that without position correction (*p* < 0.05). SSDE was different when the patient's torso center was located at the isocenter of CBCT, and when it was not. The same tendency was seen in the case of the breast. Therefore, if the center of the patient is not at the acquisition center of the CT scanner, position correction is required when estimating SSDE.

## 1 INTRODUCTION

Image‐guided radiation therapy (IGRT) has been used in many radiotherapeutic treatments and has been found beneficial in reducing setup errors and improving reproducibility.^1^ ^–^ ^4^ The modalities used in IGRT include optical body‐surface tracking, cone beam computed tomography (CBCT), X‐ray fluoroscopy, radiography, and ultrasound. Optical body‐surface tracking and ultrasound do not need to expose patients to radiation, but CBCT, fluoroscopy, and radiography do; this is an issue in IGRT because radiation therapy is often administered over a long period of time.^5^ A disadvantage of CBCT is that the radiation exposures to patients are higher than those from X‐ray fluoroscopy and optical body surface tracking systems. A disadvantage of CBCT is that the radiation exposures to patients are higher than those from X‐ray fluoroscopy and optical body surface tracking systems. For this reason, CBCT is performed in many hospitals for patients undergoing radiotherapy to improve positional accuracy. The dose delivered to the patient is smaller by CBCT than by radiotherapy. However, efforts should be made to minimize the risk of patient exposure, which requires an accurate estimation of patient dose.

In CT, the dose received by a patient depends on both the scanner's output and the patient's body size.^6^ The CT dose index (CTDI) can represent the scanner's output for a given scanner and set of acquisition parameters, but it is independent of the patient's body size. The American Association of Physicists in Medicine (AAPM) proposed the concept of size‐specific dose estimate (SSDE)^6^ that is calculated using size‐dependent factors and volumetric CTDI to estimate patient radiation dose from CT. In addition to information about the X‐ray output from the device, SSDE takes into account information about patient size. Accurate determination of the SSDE requires CTDI measured at the actual patient position because the CTDI output from the scanner would be different from the actual value if the patient's center is not at the CBCT isocenter. Therefore, in such cases, it is necessary to correct the patient's position. In diagnostic CT examinations, the center of the patient is positioned at the isocenter of the gantry to avoid image quality loss. Therefore, when measuring CTDI, a dedicated phantom is positioned at the isocenter of the CT gantry. However, in CBCT used for IGRT for breast radiotherapy, the patient's center is not positioned at the isocenter of CBCT. In other words, if SSDE is calculated based on CTDI that is measured by positioning the phantom to the isocenter of CBCT, it would overestimate or underestimate the patient radiation dose because different patient positions result in different patient dose distributions. Alvarado et al.^7^ and Rampado et al.^8^ reported on patient organ doses from CBCT for IGRT using Monte Carlo simulations and thermoluminescence dosimetry, but to this date, there are no reports on how the patient position affects patient dose in CBCT and how effective the SSDE with position correction is in the improvement of the accuracy of patient dose estimation in CBCT.

Since the dose reference point (DRP) for breast irradiation is located at the periphery of the body, the center of the patient's torso and the imaging center of the CBCT do not coincide. In such cases, the dose distribution delivered to the patient is expected to be different, and SSDE does not provide an accurate estimate of the patient's dose. Therefore, this study examined the CBCT patient dose in image‐guided radiotherapy of the breast in a case in which the patient's torso center and the CBCT imaging center did not coincide.

## 2 MATERIALS AND METHODS

### 2.1 Measurement of weighted CTDI at the CBCT isocenter

We measured weighted CTDI (CTDI_w_) at the isocenter of CBCT using the same method and conditions as those reported in our previous paper.^9^ We used an on‐board kV CBCT (Synergy XVI; Elekta Stockholm, Sweden), which is included in the Elekta Synergy accelerator. The following acquisition parameters were used for the CTDI_w_ measurements: tube voltage, 100 kV; tube current, 20 mA per frame at 5.5 frames per second; and XVI irradiation angle, from 265° to 110° in a clockwise direction. An S20 collimator was used, which yielded an axial field‐of‐view of 27 cm. No additional filter was attached (F0). The maximum diameter of the reconstruction was 270 mm, and the longitudinal X‐ray beam width (BW) was 276.7 mm. A 10‐cm ionization chamber (10 × 6‐3CT, Radcal, Monrovia, CA, USA) was used, which was connected to a digitizer (Accu‐Gold+; Radcal, Monrovia, CA, USA).

The measurement of air kerma at each point was performed thrice, and the mean value of these measurements was used. The air kerma was corrected using the following equation:

(1) $$D=K_{a}\times k_{\mathrm{TP}}\times N,$$

where *K*_a_ is the air kerma displayed on the measurement device, *D* is the measured air kerma, *k*_TP_ is the temperature atmospheric correction factor and *N* is the calibration factor of the ionization chamber. The expanded uncertainty of the calibration factor was 6%.

CTDI was measured using a 10 cm ionization chamber. In this study, the longitudinal X‐ray BW was longer than 40 mm. For this reason, CTDI 100 (CTDI_100_) was calculated using the following equation:

(2) $$\begin{array}{c} {\;\mathrm{CTDI}}_{100}=\frac{1}{{\mathrm{BW}}_{\mathrm{ref}}}\times \left(\int_{-50\;\mathrm{mm}}^{50\;\mathrm{mm}}D\left(z\right)\mathrm{dz}\right)\times \left(\frac{\left({\mathrm{CTDI}}_{\mathrm{freeair},\mathrm{BW}}\right)}{\left({\mathrm{CTDI}}_{\mathrm{freeair},\mathrm{ref}}\right)}\right), \end{array}$$

where BW_ref_ is the reference BW (20 mm), CTDI_freeair, BW_ is the CTDI measured in free air with the desired BW, and CTDI _freeair, ref_ is the CTDI measured in free air with BW_ref_.

CTDI_w_ was calculated using CTDI_100_ using the following equation:

(3) $${\mathrm{CTDI}}_{w}=\frac{1}{3}{\mathrm{CTDI}}_{100,c}+\frac{2}{3}{\mathrm{CTDI}}_{100,p},$$

where CTDI_100,c_ is the CTDI_100_ measured at the center hole of the phantom and CTDI_100,p_ is the average CTDI_100_ measured at the peripheral holes of the phantom.

### 2.2 Calculation of the position correction factor

The position correction factors were calculated using the results of a previous study.^10^ The CTDI_w_ was measured by placing the CTDI phantom from the center position (at 0 cm) to a position 16 cm away from the center along the right–left (RL) direction and from the center position to a position 7.5 cm from the center along the anterior–posterior (AP) direction to assume right breast irradiation. The relative value of CTDI_w_ was calculated using the following equation:

(4) $${\mathrm{Relative}\;\mathrm{value}\;\mathrm{of}\;\mathrm{CTDI}}_{w}\left(\%\right)=\left(\frac{{\mathrm{CTDI}}_{w}}{{\mathrm{CTDI}}_{w_{\mathrm{iso}}}}\right)\times 100,\;$$

where CTDI_w_iso_ is CTDI_w_ when the phantom is positioned at the CBCT isocenter.

The position correction factors were calculated from the relative values of CTDI_w_ using linear interpolation formulae between the two closest points of the relative values of CTDI_w_, as shown by the following equations:

(5) $$x=x_{1}+\frac{x_{2}-x_{1}}{y_{2}-y_{1}}\times \left(y-y_{1}\right),$$

(6) $$y=y_{1}+\frac{y_{2}-y_{1}}{x_{2}-x_{1}}\times \left(x-x_{1}\right),$$

where (x, y) is the point to be calculated and (x_1_, y_1_) and (x_2_, y_2_) are the nearest points to (x, y).

### 2.3 Calculation of SSDE for human phantom

For this purpose, we used an acrylic human phantom that contained human bones, which emulated the human body.

According to AAPM Report No. 220,^11^ size‐dependent factors, which are calculated from the water equivalent diameter (D_w_), are necessary for the calculation of SSDE. To calculate D_w_, the average CT number and cross‐sectional area of the axial image are required.

The average CT numbers and cross‐sectional areas inside the body contour in the axial image were obtained using the Monaco 5.11.3 (Elekta AB, Stockholm, Sweden) radiation treatment planning system (RTPS). RTPS was properly managed according to a quality assurance program defined by the guidelines of the AAPM Radiation Therapy Committee Task Group 53^12^ and the Japanese Society of Medical Physics Task Group 01.^13^ D_w_ was calculated using the following equation as described in AAPM Report No. 220^11^:

(7) $$D_{w}=2\sqrt{\left[\frac{{\bar{\mathrm{CT}\left(x,y\right)}}_{\mathrm{ROI}}}{1000}+1\right]\frac{A_{\mathrm{ROI}}}{\pi }},$$

where$\;{\bar{\mathrm{CT}(x,y)}}_{\mathrm{ROI}}$ is the average CT number within the region of interest in the axial image, and *A*_ROI_ is the area of the region of interest in the axial image.

Size‐dependent factors (*f*_size_) for the 32 cm diameter CTDI phantom were used to calculate SSDE as the right breast irradiation was selected as the target in this study. They were calculated using the obtained *D*_w_ and the approximation Equation (8) shown in AAPM Report No. 204^6^:

(8) $$\;f_{\mathrm{size}}=0.001*D_{w}^{2}-0.1059*D_{w}+3.5145.$$

SSDE was calculated by multiplying the CTDI_w_ measured at the CBCT isocenter by the size‐dependent factor. When the position correction was applied, SSDE (hereafter, corrected SSDE [SSDE_cor_]) was calculated by multiplying SSDE by the position correction factor (*f*_pos_).

(9) $$\mathrm{SSDE}={\mathrm{CTDI}}_{w}\times f_{\mathrm{size}},$$

(10) $${\mathrm{SSDE}}_{\mathrm{cor}}=\mathrm{SSDE}\times f_{\mathrm{pos}}.$$

### 2.4 Absorbed dose to the right breast according to the phantom position

For comparison between SSDE and SSDE_cor_, we examined the relationship between the position of the human phantom and the absorbed dose in the right breast. The acquisition parameters for CBCT were the same as those listed in Section 2.1. We used an acrylic human body phantom with human bones inside to emulate the human body. The absorbed doses in the right breast were measured when the center of the phantom body was placed at 9.4 cm in the AP direction and 9.9 cm in the RL direction (right breast position) from the isocenter of CBCT to align the right breast of the phantom with the isocenter of CBCT.

We used optically stimulated luminescence (OSL) dosimeters (nanoDot, Landauer, Glenwood, IL, USA) for the measurement of breast doses. A 3‐mm acrylic sheet was cut out to make the dosimeter holder, and the center of the holder was aligned with the center of the right breast. Three OSL dosimeters were inserted adjacent to each other and sandwiched between the superior and inferior phantoms. Measurements were conducted three times, and the average value was used as the final measured value. Initialization was performed by irradiating the OSL dosimeter with visible light from light‐emitting diodes for 6 h, and the values were read using a MicroStar reader (Landauer).

We calculated calibration constants for the OSL dosimeters. The following exposure parameters were used: tube voltage, 100 kV; tube current‐time product, 2 mAs; filter, F0; collimator; S20. After initialization, they were co‐irradiated with a reference ionization chamber (10 × 6‐6, Radcal, Monrovia, CA, USA), which was connected to a digitizer (Accu‐Gold+; Radcal, Monrovia, CA, USA), for intercomparison.

Absorbed doses for the right breast (*D*_RB_) were calculated from the displayed air kerma (*K*_a_) from measurement device using the following equation:

(11) $$D_{\mathrm{RB}}=\;\left(K_{a}*N_{\mathrm{OSL}}-K_{\mathrm{ai}}*N_{\mathrm{OSL}}\right)\frac{\mu_{\mathrm{en}}/\rho_{\mathrm{breast}}}{{\left(\mu_{\mathrm{en}}/\rho \right)}_{\mathrm{air}}},$$

where *K*_ai_ is the initial air kerma, *N*_OSL_ is the calibration constants of each element of the OSL dosimeter, (*μ*_en_/ρ)_breast_ is the mass energy absorption coefficient for breast (50% glandular tissue and 50% adipose tissue) and (*μ*_en_/ρ)_air_ is the mass energy absorption coefficient for air.^14^

### 2.5 Patient selection for SSDE calculation in CBCT

This observational study was approved by the ethics committee of the first author's institution, and informed consent for data publication was obtained in the form of opt‐out. For this retrospective, single‐center study, 51 patients who underwent right breast radiotherapy from July 1, 2018 to March 31, 2021, after they received breast‐conserving surgery were chosen. Patients whose DRP was not aligned with the center of the treatment beam were excluded. The mean age of the patients was 63.3&nbsp;±&nbsp;12.8 years. Body contour data and the distance from the center of the torso to the CBCT isocenter were obtained as shown in Sections 2.6–2.7.

### 2.6 Calculation of size‐dependent factors from patient CT images

In CBCT scans for IGRT, the imaging center may not coincide with the center of the patient's torso, and the body contour of the patient's cross section was not completely included owing to the limitation of the field‐of‐view. However, this problem can be solved using CT images for radiation therapy planning because they include complete body contour data of the cross‐sections of the studied patients. Thus, in this study, the D_w_ for SSDE calculation was determined from the CT images for treatment planning. The axial image containing the DRP in radiotherapy was selected for measuring the body contour of the patient. The method of calculating D_w_ was the same as that described in Section 2.3.

### 2.7 Calculation of the distribution of the patient's torso center

The difference in coordinates between the center of the patient's torso and the center of CBCT imaging was calculated using RTPS. Note that the isocenter of CBCT was consistent with the DRP in all cases. The validity of the DRP location was confirmed in all cases by a radiotherapist with more than 15 years of experience.

### 2.8 Statistical analysis

Two‐sample Student's *t*‐test was applied for the comparison between SSDE by CTDI with position correction and SSDE by CTDI without position correction. All statistical analyses were performed with SPSS (version 15.0, IBM Corporation, New York, USA). A difference was considered statistically significant when *p* < 0.05.

## 3 RESULTS

### 3.1 Position correction factor

The position correction factors ranged from 0.695 to 1.005 as the phantom's position deviated from the isocenter of CBCT along both AP and RL directions, as shown in Table 1. The position correction factors decreased as the center of the phantom moved away from the isocenter of CBCT.

**TABLE 1:** Position correction factors when the phantom's position deviated from the isocenter of the computed tomography cone beam

		Lateral direction (cm)										
		0	2	4	5	6	8	10	12	14	15	16
Anterior–posterior direction (cm)	0	1.000	1.005	0.976	0.961	0.941	0.915	0.889	0.849	0.822	0.792	0.767
	2.5	0.973	0.996	0.986	0.977	0.947	0.906	0.846	0.823	0.781	0.763	0.751
	5	0.883	0.913	0.935	0.932	0.919	0.910	0.855	0.804	0.757	0.729	0.712
	7.5	0.798	0.819	0.871	0.875	0.873	0.866	0.860	0.801	0.740	0.708	0.695

### 3.2 SSDE for the human phantom with position correction

CTDI_w_ at the isocenter of CBCT was 1.90 mGy. The sizes of the phantom used in this measurement were 23.17 cm in the AP direction and 26.12 cm in the RL direction and resulted in size‐dependent factors equal to 1.51. The position correction factor was 0.85 and was calculated from the coordinates of the dosimetry points in the right breast and the isocenter of CBCT. Therefore, SSDE_cor_ and SSDE values for the human phantom were 2.34 and 2.75 mGy, respectively. SSDE_cor_ was 15% lower than SSDE.

### 3.3 Absorbed doses to the right breast in the human body phantom

The calibration constants of the OSL dosimeters ranged from 0.828 to 0.969 (mean, 0.892; standard deviation, 0.036).

The absorbed D_RB_ were 2.23 ± 0.14 mGy at the phantom center position and 1.99 ± 0.08 mGy at the right breast position. The absorbed dose at the right breast position was 89.2% of the phantom center position.

### 3.4 Size‐dependent factors and distance between the CBCT isocenter and the center of the patient's torso

In the 51 patients, the mean and standard deviation of size‐dependent factors was 1.51 ± 0.14 (minimum, 1.33; maximum, 1.88). Size‐dependent factors were inversely related to body weight.

**FIGURE 1:**
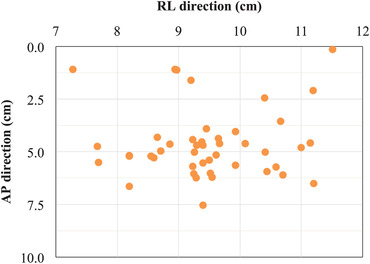
Distribution of the coordinates of the center of the patient's torso. AP, anterior–posterior direction; RL, right–left direction

Figure 1 shows the distribution of the coordinates obtained. In the AP direction, the maximum and minimum deviations of the torso center from the CBCT imaging center were 7.53 and 0.14 cm, respectively. In the RL direction, the maximum and minimum deviations were 11.51 and 7.28 cm, respectively. The mean deviations were 9.42 ± 0.95 cm in the AP direction and 4.67 ± 1.69 cm in the RL direction.

### 3.5 Calculation of SSDE and position correction

**FIGURE 2:**
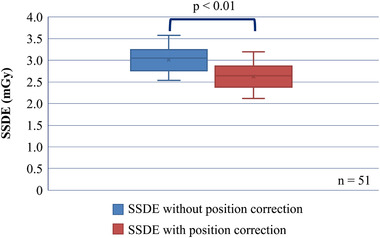
Side‐specific dose estimate (SSDE) with position correction and SSDE without position correction. Error bars represent standard deviation.

Figure 2 shows the relationship between SSDE_cor_ and SSDE. The mean and standard deviation of SSDE_cor_ and SSDE were 2.54 ± 0.29 and 2.92 ± 0.30 mGy, respectively, and SSDE_cor_ was significantly lower than SSDE. The median patient shift was 9.40 cm in the RL direction and 4.95 cm in the AP direction. The SSDE decreased by 13.2% for patients with a shift of 9.4 cm in the RL direction and 4.7 cm in the AP direction. It was shown that positional correction significantly reduced SSDE, and it decreased as the center of the patient's torso moved away from the isocenter of CBCT.

## 4 DISCUSSION

In this study, comparing SSDE_cor_ and SSDE in the phantom, it was shown that SSDE decreased with position correction. Alvarado et al.^7^ reported that in CBCT in IGRT, the breast dose was 10% to 23% lower than that when the center of the torso was placed at the isocenter of CBCT, and when the center of the breast was placed at the isocenter of CBCT. The results of the absorbed doses by OSL measurements showed that the SSDE and right breast doses yielded similar changes. It is considered that this was due to the bias of the dose distribution in the axial plane because X‐rays were irradiated to the subject from partial directions (265–110° in a clockwise direction). In other words, when the isocenter of the CBCT was located at the DRP in the right breast, the subject was farther away from the direction of X‐ray incidence than when the isocenter of the CBCT was located at the center of the subject. Therefore, it is considered that the dose decreased as the subject moved away from the X‐ray source.

In the clinical study, we calculated SSDE using the body contour data of the patients who received right breast radiotherapy after correcting the position and found a significant difference between SSDE and SSDE_cor_. In addition, we showed that SSDE decreased as the distance between the center of the torso and the CBCT imaging center increased. This result was similar to the result of the phantom study. Therefore, when the center of the patient's torso and the isocenter of the CBCT do not coincide, position correction is recommended to be used to calculate SSDE based on the actual position of the patient.

Note that this study is associated with several limitations. There is room for investigating the appropriateness of applying CTDI measurement for CBCT dosimetry. In this study, CTDI was measured using the method prescribed by International Electrotechnical Commission.^9^ However, Amer et al.^15^ proposed the use of the cone‐beam DI (CBDI), whereas Kim et al.^16^ compared several suitable measurement methods for CBCT, including CBDI. AAPM TG111^17^ proposed the dose measurement method in conjunction with the use of a phantom that had a size of 45 cm in the long direction and an ionization chamber dosimeter of 0.6 cm^3^. According to Buckley et al.,^18^ conventional CTDI underestimates the dose to patients when the BW is wide, and the method proposed in TG111 is the best estimation of the dose in the center of a PMMA phantom in CBCT because the scattering profile in the body axis direction is taken into account.^17^ In addition, the results in the present study were obtained using a single device and a single combination of acquisition parameters, but the values may be different in the cases of other devices. However, if the center of the subject is located away from the CBCT isocenter, we expect to see a similar trend. In this study, SSDE was calculated only for patients who received radiation to the right breast, but we think that similar methods can be applied to other sites.

## 5 CONCLUSIONS

We showed that SSDE was different when the patient's torso center was located at the isocenter of CBCT and when it was not. The same tendency was observed with the right breast doses. The median movement in this study was 9.4 cm from the isocenter in the RL direction and 4.7 cm in the AP direction. In this case, SSDE decreased by 13.2%. Therefore, if the center of the patient is not at the acquisition center of CBCT, position correction is required when estimating SSDE.

## AUTHOR CONTRIBUTION

*Conceptualization, data curation, investigation, visualization, and writing – original draft*: Hiroyuki Ueno. *Supervision, writing – review and editing*: Kosuke Matsubara. *Writing‐ reviewing and editing*: Masato Hizume and Sayuri Bou.

## CONFLICT OF INTEREST

The authors declare no conflict of interest.

## ETHICS STATEMENT

This study was approved by the ethics committee of the first author's institution (reference number: 2021 2–21), and informed consent for data publication was obtained in the form of opt‐out.

## Data Availability

The data that support the findings of this study are available from the corresponding author upon reasonable request.
